# A Case in Practice

**Published:** 1882-11-20

**Authors:** G. A. Cook

**Affiliations:** Georgia


### Southern Medical Record:

EDITORS:

T. S. POWELL, M.D. W. T. GOLDSMITH, M.D. R. C. WORD, M.D.

*R. C. WORD, M.D., Managing Editor.*

☞All Communications and Letters on Business connected with the RECORD must be addressed to the Managing Editor.

VOL. XII. ATLANTA, GA., NOV. 20, 1882. NO. 11.

### ORIGINAL AND SELECTED ARTICLES.

### A CASE IN PRACTICE.

BY G. A. COOK, M. D., OF GEORGIA.

Mr. A. M., æt. 33. Married gentleman, of moral habts, occupation is that of an accountant. I have known him for the past twelve years. Very early in the morning, on the 7th day of October, I was called to see the case, reported to me, as being very sick. On my arrival, the patient told me he had to go to stool, and during the passage of fæcal matter he felt something hook in his rectum, which stopped the act of defecation, and which gave him very severe pain, from which he was at the time suffering. Upon examination, I found, as he had stated, a hard foreign body, flat and of triangular shape, the sides of which were an inch in length, hooked in the mucous membrane of the rectum, about an inch above the sphincter. Not expecting to see anything appertaining to surgery, I had nothing but nature's forceps to work with, I succeed in disengaging the foreign body, which proved to be a bone of the above shape and dimensions. This bone having entered his alimentary canal some time since—probably a year or more ago, judging from the account of the patient and appearance of the bone, it having undergone a considerable discoloration, with marks on its flat sides and edges, as evidence that the alimentary juices had faithfully tried to put it in a condition for assimilation. This foreign substance having traversed the extent of the alimentary canal, when the only evidence of its being there was manifested on the morning of the 7th of October. The only annoyance during the extensive travel of this bone was some slight abrasions of the mucous membrane, made with the sharp edges of the bone by the resistance of the sphincter muscles. I would ask, is it not something remarkable?

